# Correlative Analysis Between Adverse Events of Preoperative Chemotherapy and Postoperative Complications of Gastric Cancer

**DOI:** 10.3389/fsurg.2021.768243

**Published:** 2021-12-02

**Authors:** Zhouqiao Wu, Yiding Wang, Shiyang Hou, Qi Wang, Bailong Li, Xiangji Ying, Shuangxi Li, Ziyu Li, Jiafu Ji

**Affiliations:** Key Laboratory of Carcinogenesis and Translational Research (Ministry of Education), Gastrointestinal Cancer Center, Peking University Cancer Hospital and Institute, Beijing, China

**Keywords:** postoperative complications, adverse events, preoperative chemotherapy, gastric cancer, propensity score matching

## Abstract

**Background and Objectives:** This study aims to explore the safety of preoperative chemotherapy and clarify whether preoperative chemotherapy with oxaliplatin + S-1 (SOX) regimen and its adverse events are associated with higher risks of postoperative complications.

**Methods:** We included consecutive patients with gastric cancer who underwent gastrectomy in our department between July 1 2018, and January 31 2020. Patients with preoperative SOX regimen chemotherapy were included in the analysis.

**Results:** In the 343 included patients, 77 cases underwent preoperative chemotherapy. In total, surgical complications were found in 117 patients (34.1%), and there was no significant difference between the patients with and without preoperative chemotherapy before and after propensity score matching (*p* > 0.05, respectively). Multivariate analysis showed that preoperative comorbidities (*p* = 0.026) and the preoperative cT4b (*p* = 0.028) were independent risk factors in postoperative complications. In patients with preoperative chemotherapy, neither the occurrence of adverse events nor their severity was associated with postoperative complications (*p* > 0.05). However, the patients who received five to six cycles were more prone to postoperative complications than those who received three to four cycles (62.5 vs. 27.9%, OR = 4.306, 95% Cl = 1.282–14.464, *p* = 0.018).

**Conclusions:** Occurrence of postoperative complications was not influenced by preoperative SOX chemotherapy. However, increased cycles of chemotherapy may lead to higher incidence of postoperative complications.

## Introduction

According to the WHO global cancer data, over one million new cases of gastric cancer worldwide have occurred annually in the recent years, ranking the sixth malignant tumors in the world in terms of morbidity and the second in terms of mortality (1). Although surgical resection remains the mainstream of gastric cancer treatment, chemotherapy offers additional survival benefit (2–4), and thus has been widely implemented in perioperative settings. Preoperative chemotherapy for gastric cancer, first pioneered by Schirren et al. and many other Western colleagues, has gained increasing popularity in the recent years (5). In addition to the survival benefit, the advantages of preoperative chemotherapy mainly include down staging, a better R0 resection rate, early control of micro-metastasis, better tolerance of adverse events, and better evaluation of therapy effect compared to postoperative settings (6).

With the accumulating evidence and guidelines suggestions, concerns remain with regard to its wide applications. For example, deterioration of physical conditions of patients caused by adverse events of preoperative chemotherapy may lead to the decrease of the ability of the patients to tolerate surgery and increase the occurrence of postoperative complications. Very high complication rates varying between 20 and 50% were reported in patients with preoperative chemotherapy, which seems disturbing for its further application because surgical complications may not only cause direct morbidity and even mortality after operation but also diminish the survival benefits of preoperative therapy (7–9). Moreover, with many international studies on neoadjuvant chemotherapy for gastric cancer, there is still no consensus with regard to the optimal regimens and therapy cycles. Although it is recommended by many Western guidelines, its application in East Asia, where most gastric cancer occurs, is still limited, partly due to lack of evidence. Our center is one of the leading centers attempting neoadjuvant chemotherapy (NACT) in China, and the oxaliplatin + S-1 (SOX) regimen is the most frequently used. To this end, we analyzed our prospectively maintained database to determine the influence of preoperative chemotherapy with SOX regimen on the perioperative safety. This study aims to determine whether preoperative SOX chemotherapy increases postoperative complications, and whether preoperative chemotherapy adverse events and its duration increase postoperative complications.

## Methods

### Patients

This is a retrospective analysis of our prospectively maintained clinical database. We included consecutive patients with gastric cancer who underwent gastrectomy in our department between July 1, 2018 and January 31, 2020. Only adult patients with histologically proven gastric cancer in biopsy were included, and the patients who met any of the following criteria were excluded from the analysis: remnant gastric cancer; diagnosed with other malignant diseases within 5 years; urgent operation (e.g., bleeding, perforation, and obstruction); insufficient safety data (e.g., received chemotherapy in other hospitals); other diseases requiring synchronous surgery; preoperative treatment with HIPEC (Hyperthermic Intraoperative Peritoneal Chemotherapy); preoperative chemotherapy with other regimen than SOX; and preoperative treatment with targeted therapy or immunotherapy.

### NACT and Perioperative Treatment

In our center, clinical staging of gastric cancer includes endoscopy (with biopsy), endoscopic ultrasound for early cases, abdominal enhanced CT, chest plain scan or enhanced CT scan, cervical lymph node ultrasound, and positron emission tomography (PET) scan (in selected patients when necessary). Laparoscopic exploration and abdominal cytology were conducted before NACT to further determine the peritoneal and cytological status. All staging was determined based on the 8th edition of the AJCC Cancer Staging Manual by the American Joint Committee on Cancer and International Union Against Cancer (10).

Our center is one of the first centers who pioneered NACT in gastric cancer in China. NACT is discussed with all locally advanced patients as a treatment option before surgical resection. A shared decision would be made and carried out afterwards. We only included the patients with SOX regimen to reach a better consistency in this study, which enables further analysis into the influence of adverse events (AE) and cycles to surgical safety. For those who chose NACT, SOX regimen, i.e., intravenous oxaliplatin (Day 1) plus oral S-1 (Days 1–14), followed by 1-week rest, was administered, and tumor response was evaluated by enhanced CT in every two to three cycles. During the treatment, clinical data are prospectively collected, including the dose modification due to chemotherapy related adverse events, hematologic and biochemical parameters, chemotherapy tolerance, and disease progression. After NACT, a standard gastrectomy would be performed in accordance with the Japanese Gastric Cancer Treatment Guidelines (11). As we have previously demonstrated, the safety of laparoscopic gastrectomy after NACT (12), both open and laparoscopic approaches are optional as per disease condition or choice of a doctor.

### Data Collection

For the purpose of this study, we collected the following data for analysis. The baseline characteristics of the patients include gender, age, height, weight, BMI, city, NRS-2002, ECOG, KPS, ASA score, preoperative comorbidity, smoking history, drinking history, and family history.

The surgical characteristics include cT stage, cN stage, cM stage, surgical procedure, reconstruction approach, operating time, tumor location, resection range, multiorgan excision, lymph node dissection range, and surgical radicalness.

Toxicity and adverse events during chemotherapy were collected and evaluated by case report form (CRF) in nine aspects, which were classified according to Common Terminology Criteria for Adverse Events (CTCAE) v5 (13).

Severe adverse events (SAEs) were CTCAE grade ≥ III. The following variables were obtained during every cycle of preoperative chemotherapy: alkaline phosphatase, white blood cell count, glutamic-pyruvic transaminase (ALT), glutamic oxalacetic transaminase (AST), hemoglobin concentration, lymphocyte count, neutrophil count, blood platelet count, prognostic nutritional index, and serum creatinine.

Postoperative complications are prospectively collected by the attending physicians, and researchers majored in postoperative complications are in charge of the quality control. Case discussions are held weekly to further verify the complications during their stay in hospital and treatment after surgery. Complications are categorized by the Expert Consensus on the Diagnostic Registration Criteria for Postoperative complications of Gastrointestinal Cancer surgery in China (2018 edition). Severe complications were definite as Clavien-Dindo Grade ≥ III.

### Propensity-Score Matching (PSM) Analysis

The included patients were divided into the NACT group and the control group in accordance with the treatment. Given the fact that NACT is applied to locally advanced cases and many relatively early-stage cases are in the control group, PSM is used to balance the patient characteristics between the groups. The patients were individually matched between those with and without preoperative chemotherapy using the nearest-neighbor-matching method, where the matching ratio is one is to one, and the cut-off value was 0.02. The matching variables are selected based on the factors that usually influence the choice of NACT (age, clinical stage, and preoperative comorbidity) and our previous research with regard to the risk factors of postoperative complications (tumor location, clinical stage, and preoperative comorbidity). The propensity-score matching analysis was performed with SPSS^®^ software package 26.0 (SPSS Inc., Chicago, IL, USA).

### Statistical Analysis

Categorical data were compared with the use of the *x*^2^ test or Fisher's exact test. Continuous variables that did not coincide with normal distribution were transformed into hierarchical data and compared with the use of the *x*^2^ test or Fisher's exact test. Complication risk factors were evaluated by binary data logistic regression analysis. Variables with a univariate *p* < 0.10 were considered in a multivariate analysis. A *p* < 0.05 (two-sided) was considered significant. The Hazard ratio and 95% CI will be generated by SPSS^®^ software package 26.0 (SPSS Inc., Chicago, IL, USA).

## Results

### Baseline Characteristic and Surgical Complication

**Figure 1:**
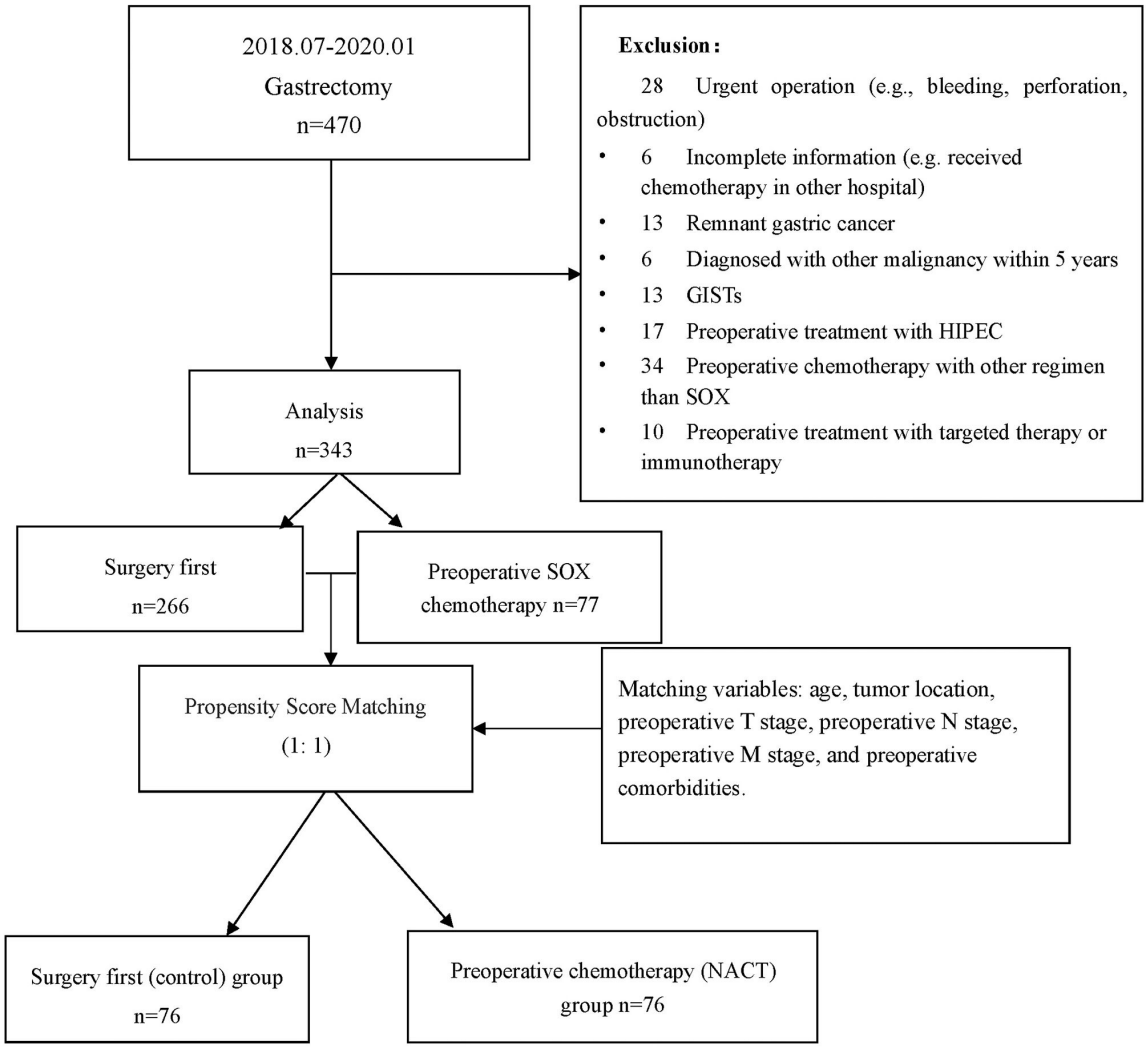
Flowchart for patient inclusion and exclusion. *GIST, gastrointestinal stromal tumors; HIPEC, hyperthermic introperitoneal chemotherapy.

The study collected a total of 470 patients who underwent gastrectomy from July 1, 2018 to January 31, 2020. There were 127 cases excluded for various reasons (see Figure 1), resulting in a total of 343 patients, including 228 men (66.5%) and 115 women (33.7%) included for analysis. The patient characteristics are listed in Table 1 (for detailed information, see Supplementary Table 1). There were 77 cases in the preoperative chemotherapy group and 266 cases in the direct operation group. There was no statistical difference in the general clinical information between the two groups—(see Table 1).

**Table 1:** Clinical characteristics of the two groups before and after PSM.

**Variable**	**Before matching**		***P* Value**	**After Matching**		***P* Value**
	**Control** **(*N* = 266)**	**NACT** **(*N* = 77)**		**Control'** **(*N* = 76)**	**NACT'** **(76)**	
Male/Female	170/96	58/19	0.062	53/23	57/19	0.468
Age			0.729			0.134
<60 years	115 (43.2%)	35 (45.5%)		51 (67.1%)	42 (55.3%)	
≥60 years	151 (56.8%)	42 (54.5%)		25 (32.9%)	34 (44.7%)	
Preoperative comorbidities			0.194			0.345
No	191 (71.8%)	61 (79.2%)		55 (72.4%)	60 (78.9%)	
Yes	75 (28.2%)	16 (20.8%)		21 (27.6%)	16 (21.1%)	
Multiorgan excision			0.170			>0.999
No	245 (92.1%)	67 (87.0%)		66 (86.8%)	66 (86.8%)	
Yes	21 (7.9%)	10 (13.0%)		10 (13.2%)	10 (13.2%)	
Resection range			<0.001			0.282
Distal	175 (65.8%)	35 (45.5%)		42 (55.3%)	34 (44.7%)	
Proximal	25 (9.4%)	5 (6.5%)		2 (2.6%)	5 (6.6%)	
Total	66 (24.8%)	37 (48.1%)		32 (42.1%)	37 (48.7%)	
Pre-operative T stage			<0.001			0.107
1	55 (20.7%)	0 (0%)		1 (1.3%)	0 (0.0%)	
2	33 (12.4%)	2 (2.6%)		2 (2.6%)	2 (2.6%)	
3	79 (29.7%)	34 (44.2%)		21 (27.6%)	34 (44.7%)	
4a	80 (30.1%)	36 (46.8%)		41 (53.9%)	35 (46.1%)	
4b	19(7.1%)	5(6.5%)		11(14.5%)	5(6.6%)	
Pre-operative N stage			<0.001			0.962
0	120 (45.1%)	10 (13.0%)		8 (10.5%)	10 (13.2%)	
1	75 (28.2%)	17 (22.1%)		18 (23.7%)	17 (22.4%)	
2	46 (17.3%)	37 (48.1%)		37 (48.7%)	37 (48.7%)	
3	25 (9.4%)	13 (16.9%)		13 (17.1%)	12 (15.8%)	
Preoperative M stage			0.418			0.240
0	246 (92.5%)	69 (89.6%)		63 (82.9%)	68 (89.5%)	
1	20 (7.5%)	8 (10.4%)		13 (17.1%)	8 (10.5%)	
Reconstruction approach			0.003			0.415
Billroth I	23 (8.6%)	1 (1.3%)		3 (3.9%)	1 (1.3%)	
Billroth II	153 (57.5%)	37 (48.1%)		40 (52.6%)	36 (47.4%)	
Roux-en-Y and others	90 (33.8%)	39 (50.6%)		33 (43.4%)	39 (51.3%)	
Lymph node dissection range			0.248			>0.999
D2	248 (93.2%)	76 (98.7%)		74 (97.4%)	75 (98.7%)	
D1+	13 (4.9%)	1 (1.3%)		1 (1.3%)	1 (1.3%)	
D1	5 (1.9%)	0 (0.0%)		1 (1.3%)	0 (0.0%)	
Surgical radicalness			0.256			0.037
R0	255 (95.9%)	77 (100.0%)		70 (92.1%)	76 (100.0%)	
R1	9 (3.4%)	0 (0.0%)		6 (7.9%)	0 (0.0%)	
R2	2 (0.8%)	0 (0.0%)		0 (0.0%)	0 (0.0%)	
Tumor location			0.115			0.377
L	141 (53.0%)	32 (41.6%)		41 (53.9%)	31 (40.8%)	
EGJ	40 (15.0%)	22 (28.6%)		15 (19.7%)	22 (28.9%)	
U	28 (10.5%)	8 (10.4%)		9 (11.8%)	8 (10.5%)	
M	55 (20.7%)	14 (18.2%)		9 (11.8%)	14 (18.4%)	
Total gastric	2 (0.8%)	1 (1.3%)		2 (2.6%)	1 (1.3%)	
Operating time			0.007			0.081
<180 min	111 (41.7%)	19 (24.7%)		29 (38.2%)	19 (25.0%)	
≥180 min	155 (58.3%)	58 (75.3%)		47 (61.8%)	57 (75.0%)	
Complications			0.196			0.504
No	180 (67.7%)	46 (59.7%)		49 (64.5%)	45 (59.2%)	
Yes	86 (32.3%)	31 (40.3%)		27 (35.5%)	31 (40.8%)	
Complication stratification			0.395			0.669
No complications	180 (67.7%)	46 (59.7%)		49 (64.5%)	45 (59.2%)	
I/II	75 (28.2%)	28 (36.4)		23 (30.3%)	28 (36.8%)	
III/IV	11 (14.5%)	3 (3.9%)		4 (5.3%)	3 (3.9%)	

The detailed perioperative characteristics of the included patients are listed in Supplementary Table 2. Before PSM, the chemotherapy group had shown significantly longer operating time and higher clinical T and N stage when compared with the surgery group (*p* < 0.05, respectively, Table 1). It also had higher proportion of EGJ cases, which resulted in the significant different resection ranges and reconstruction approaches between the groups (Table 1).

Complications were found in 117 patients (34.1%) after surgery, and there was no significant difference between the groups before PSM. Logistic regression was conducted, and multivariate analysis showed that preoperative comorbidities (*p* = 0.026) and the preoperative T stage cT4b (*p* = 0.028) as independent risk factors in postoperative complications (Supplementary Tables 3.1, 3.2). Preoperative chemotherapy is not a risk factor in postoperative complications.

### Surgical Complication After PSM

Given the significant difference between the NACT and surgery groups, PSM was conducted. Covariates that influence choice of NACT (such as age and clinical TNM stage) and postoperative complication (such as preoperative comorbidity, clinical TNM stage, and tumor location) were included in the propensity matching score, with a 1:1 matching, and the Caliper value was set to 0.02. After matching, a total of 152 patients were included in further analysis, including 76 in the control' group and 76 in the NACT group.

After matching, the complication rate was 35.5% in the surgery group and 40.8% in the NACT group. The majority of complications were Grades I and II, and there was no statistical difference between the groups in the total number of complications and the different grades of complications (Table 1).

### NACT Adverse Events and Cycles and Surgical Complication

All 77 patients who received preoperative chemotherapy recorded adverse events (see Supplementary Table 4). There were 22 cases (28.6%) of AEs with Grade 3 or higher, including six cases (7.8%) of blood/marrow system, seven cases (9.1%) of systemic symptoms, nine cases (11.7%) of gastrointestinal system, one case of skin (1.3%), two cases (2.6%) of metabolic/laboratory examination.

In total, 31 of 77 cases (40.26%) had postoperative complications, and 3 cases of major complications (3.90%) (Table 2). Detailed analysis showed that there was no significant association between any of the nine aspects of AE and occurrence of complication (Supplementary Table 5). There was no significant difference in patients with Grades 2, 3, and 4 when compared with the Grade 1 cases, respectively (Table 3). The majority of the patients (43 cases) had three-to-four-cycle NACT. Compared to that, there was no statistical difference in the incidence of postoperative complications among the patients who received one to two, seven to eight cycles. However, the patients who received five to six cycles were more prone to postoperative complications than those who received three to four cycles (*p* = 0.018) (Table 3).

**Table 2:** Classification of postoperative complications in the preoperative chemotherapy group^*^.

**Classification**	**CD Classification**			
	**I**	**II**	**III**	**IV**
Unexplained fever		6		
Reflux esophagitis		1		
Abdominal effusion or abscess formation		3	1	
Intra-abdominal hemorrhage				1
Diarrhea	1			
Lymphatic leakage	2	2		
Urinary tract infection		2		
Chylotrhea	1			
Anastomotic leakage	1			
Gastrointestinal bleeding	1			
Arrhythmia	1			
Pleural effusion		1		
Pancreatic leakage		7	1	
Others	3			

* *Some patients have more than one complication*.

**Table 3:** Association between complications and preoperative adverse events.

**Variable**	**No complications (*N* = 46)**	**Complications (*N* = 31)**	**OR (95%CI)**	***P* Value**
Most serious adverse event grade				
1	10 (21.7%)	3 (9.7%)	Ref	
2	24 (52.2%)	18 (58.1%)	2.500 (0.600–10.422)	0.208
3	11 (23.9%)	8 (25.8%)	2.424 (0.500–11.761)	0.272
4	1 (2.2%)	2 (6.5%)	6.667 (0.437–101.732)	0.172
Cycles				
3–4	31 (67.4%)	12 (38.7%)	Ref	
1–2	8 (17.4%)	7 (22.6%)	2.260 (0.672–7.608)	0.188
5–6	6 (13.0%)	10 (32.3%)	4.306 (1.282–14.464)	0.018
7–8	1 (2.2%)	2 (6.5%)	5.167 (0.428–62.393)	0.196

## Discussion

For surgeons, surgical safety prioritizes all surgical and therapeutic innovations. This analysis of our prospectively maintained database revealed that occurrence of postoperative complications was not influenced by preoperative chemotherapy with the SOX regimen but by preoperative comorbidity, tumor location, clinical stage, and resection range (including multiorgan excision). Neither the occurrence of chemotherapy adverse events nor the severity of them was associated with postoperative complications. However, our study also suggested that increased cycles of preoperative chemotherapy may lead to higher incidence of postoperative complications. We believe our data are suggestive to doctors during therapy selection for patients with gastric cancer.

The patient characteristics between the groups were imbalanced. There was no patient in the chemotherapy group, but 53 in the control group were in stage of T1N0M0. The percentage of T4a and T4b was accounted for 53.3% in the NACT group, significantly higher than that of 37.8% of the patients in the control group. Similar results were also found in the analysis of the preoperative N staging. These results were expected because NACT is only suggested to locally advanced cases, especially those in the stage of T3-4N+ as a therapeutic option in addition to the surgery-first strategy. The significant differences in clinical staging resulted in the subsequent differences in the resection range, duration of operation, and anastomotic methods. Many of the imbalanced factors, such as tumor location, operating time, and combined visceral resection, were identified as independent risk factors of complications, which are often reported in the literature as well (14–17). Given that, propensity score matching is necessary for our study to reduce the selection bias. Due to the retrospective nature of our study, these efforts were made to reach the best confidence of our conclusions (18).

After PSM, a higher rate of R0 resection was also found in the chemotherapy group, which was in accordance with the literature as one of the advantages of preoperative chemotherapy. However, longer duration of operation was still reported in the NACT group even after matching. Similar results had also been reported by Ramachandra (19) and Yuan (15), and there are at least two major reasons. First, due to lack of solid evidence in this regard, the surgical resection range, especially the lymphadenectomy resection range, is usually determined by the naïve clinical stage but not the yielded clinical stage even in cases with good remission (CR or PR) in our practice. This may cause longer surgical duration in the patients with NACT. Moreover, preoperative chemotherapy may lead to tissue adhesion, edema, and fibrosis, prolonging the surgical process. In general, the prolonged surgical process indicates higher incidence of postoperative complications. However, our results did not turn out that way, confirming the safety of preoperative chemotherapy. Our ongoing study is to further investigate whether there is a causal association between those tissue changes and complications.

The complication rate after gastrectomy was 34.1% in our study, most of which were categorized into Grade I/II (30.0%), while the rate of Grade III/IV was 4.1%. Such data are comparable to many prospective trials. After years of consecutively advocating early and strict registration of surgical complications, our center, together with many other major centers in China, reached reliable reported complication rates that are comparable to the literature (17, 20–22). The high rate of reported Grade I/II and the low rate of Grade III/IV complications indicate a good complication registration rate, and also suggest early detection, early diagnosis, and early intervention may improve the complication outcomes. In our study, postoperative complications were categorized according to the Expert Consensus on the Diagnostic Registration Criteria for Postoperative complications of Gastrointestinal Cancer surgery in China (2018 edition). It specifies how to register and grade complications, which was beneficial for the registration of early complications.

Similar rates of complications were found in the two groups regardless the preoperative therapy; our data confirm the safety of SOX as a choice of preoperative treatment strategy. Adverse events are very common, actually almost inevitable, during preoperative chemotherapy based on our data and the literature. The rate of severe adverse events reported in our study (28.6%) was lower than some previous reports and comparable to the others (23, 24). This is partially because the patients receiving preoperative chemotherapy are usually at better physical condition and better quality of life than those who receive after surgery, which may also lead to better tolerance of those adverse events. But more importantly, the SOX regimen has much less dose intensity when compared with the regimens applied in other trials, such as docetaxel, cisplatin, and S-1 (23), or docetaxel, oxaliplatin, and capecitabine (25). To this end, it is, therefore, important to emphasize that it is yet to conclude the influence of preoperative chemotherapy with much higher intensity to the surgical safety.

To date, the optimal cycles for gastric cancer preoperative chemotherapy are yet to be determined. Although, usually, two to three cycles are adopted nowadays, the COMPASS trial suggested the higher rate of pathological complete response in more cycles, which showed survival benefits in our previous analysis (26). It was unexpected to find more cycles of preoperative chemotherapy were associated with increased incidence of postoperative complications. The possible theory is the accumulation of chemotherapy toxicity due to the prolonged cycles. This is also partially supported by the higher rate of complications in the patients who underwent seven-to-eight-cycle preoperative chemotherapy. Of course, the incidence of complications in the patients with one to two cycles of chemotherapy was also slightly higher than that in the patients with three to four cycles of chemotherapy. But these patients are often the ones who were intolerant or had no response to preoperative chemotherapy. Although analysis with larger sample-sized database may further reveal the influence of preoperative chemotherapy to surgery, out data seem to indicate that the highest grade of adverse event may not properly represent the chemotherapy toxicity, and other parameters are still in request in this regard.

In conclusion, our data suggest that occurrence of postoperative complications was not influenced by preoperative chemotherapy with the SOX regimen but by preoperative comorbidity, tumor location, clinical TNM staging, and resection range (including multiorgan excision). Neither the occurrence of adverse events nor their severity during preoperative chemotherapy was associated with postoperative complications. However, increased cycles of preoperative chemotherapy may lead to a higher incidence of postoperative complications. We believe our data are suggestive to doctors during therapy selection for patients with advanced gastric cancer.

## Data Availability Statement

The original contributions presented in the study are included in the article/Supplementary Material, further inquiries can be directed to the corresponding author.

## Ethics Statement

The studies involving human participants were reviewed and approved by Peking University Cancer Hospital Ethics Committee (2016YJZ32). The patients/participants provided their written informed consent to participate in this study.

## Author Contributions

ZW and YW wrote the manuscript and researched the literature. ZW, YW, and SH contributed to performed bioinformatics analysis, material preparation, figures, and tables. SH, QW, BL, XY, and SL collected the clinical data and drafted the work. ZL and JJ supervised the study and contributed to the manuscript revision. All authors have read and approved the final manuscript.

## Funding

This study was funded by the National Key Technology Research and Development Program of the Ministry of Science and Technology of China (D171100006517004), Bethune Charitable Foundation (to ZW), and Beijing Municipal Administration of Hospitals' Youth Program (QML20191103).

## Conflict of Interest

The authors declare that the research was conducted in the absence of any commercial or financial relationships that could be construed as a potential conflict of interest.

## Publisher's Note

All claims expressed in this article are solely those of the authors and do not necessarily represent those of their affiliated organizations, or those of the publisher, the editors and the reviewers. Any product that may be evaluated in this article, or claim that may be made by its manufacturer, is not guaranteed or endorsed by the publisher.

## References

[1] Bray F Ferlay J Soerjomataram I Siegel RL Torre LA Jemal A. Global cancer statistics 2018: GLOBOCAN estimates of incidence and mortality worldwide for 36 cancers in 185 countries. CA Cancer J Clin. (2018) 68:394–424. 10.3322/caac.21492 30207593

[2] Kanhere H Goel R Finlay B Trochsler M Maddern G. Radical gastrectomy: still the cornerstone of curative treatment for gastric cancer in the perioperative chemotherapy era-a single institute experience over a decade. Int J Surg Oncol. (2018) 2018:9371492. 10.1155/2018/9371492 29568650 PMC5820646

[3] Stahl M Walz MK Stuschke M Lehmann N Meyer HJ Riera-Knorrenschild J. Phase III comparison of preoperative chemotherapy compared with chemoradiotherapy in patients with locally advanced adenocarcinoma of the esophagogastric junction. J Clin Oncol. (2009) 27:851–6. 10.1200/JCO.2008.17.0506 19139439

[4] Zhang ZX Gu XZ Yin WB Huang GJ Zhang DW Zhang RG. Randomized clinical trial on the combination of preoperative irradiation and surgery in the treatment of adenocarcinoma of gastric cardia (AGC)–report on 370 patients. Int J Radiat Oncol Biol Phys. (1998) 42:929–34. 10.1016/S0360-3016(98)00280-6 9869212

[5] Schirren R Reim D Novotny AR. Adjuvant and/or neoadjuvant therapy for gastric cancer? A perspective review. Ther Adv Med Oncol. (2015) 7:39–48. 10.1177/1758834014558839 25553082 PMC4265092

[6] Li SX Seo SH Choi YY Nakagawa M An JY Kim HI. Correlation analyses between pre- and post-operative adverse events in gastric cancer patients receiving preoperative treatment and gastrectomy. BMC Cancer. (2016) 16:29. 10.1186/s12885-016-2066-y 26786480 PMC4717569

[7] Cunningham D Allum WH Stenning SP Thompson JN Van de Velde CJ Nicolson M. Perioperative chemotherapy versus surgery alone for resectable gastroesophageal cancer. N Engl J Med. (2006) 355:11–20. 10.1056/NEJMoa055531 16822992

[8] Slagter AE Jansen EPM van Laarhoven HWM van Sandick JW van Grieken NCT Sikorska K. CRITICS-II: a multicentre randomised phase II trial of neo-adjuvant chemotherapy followed by surgery versus neo-adjuvant chemotherapy and subsequent chemoradiotherapy followed by surgery versus neo-adjuvant chemoradiotherapy followed by surgery in resectable gastric cancer. BMC Cancer. (2018) 18:877. 10.1186/s12885-018-4770-2 30200910 PMC6131797

[9] Ashraf N Hoffe S Kim R. French FNCLCC/FFCD 9703 study. Oncologist. (2014) 19:431. 10.1634/theoncologist.2013-0456 24729607 PMC3983828

[10] Oh DY Bang YJ. Adjuvant and neoadjuvant therapy for gastric cancer. Curr Treat Options Oncol. (2013) 14:311–20. 10.1007/s11864-013-0238-4 23686725

[11] Japanese Gastric Cancer Association. Japanese gastric cancer treatment guidelines 2018 (5th edition). Gastric Cancer. (2020) 14:101–12. 10.1007/s10120-011-0041-5 32060757 PMC7790804

[12] Li Z Shan F Ying X Zhang Y. E JY Wang Y. Assessment of laparoscopic distal gastrectomy after neoadjuvant chemotherapy for locally advanced gastric cancer: a randomized clinical trial JAMA Surg. (2019) 154:1093–101. 10.1001/jamasurg.2019.3473 31553463 PMC6763995

[13] Common Terminology Criteria for Adverse Events (CTCAE). US Department of Health and Human Services. National Institutes of Health National Cancer Institute. (2017).

[14] Wang X Yao Y Qian H Li H Zhu X. Longer operating time during gastrectomy has adverse effects on short-term surgical outcomes. J Surg Res. (2019) 243:151–9. 10.1016/j.jss.2019.05.021 31176285

[15] Yuan P Wu Z Li Z Bu Z Wu A Wu X. Impact of postoperative major complications on long-term survival after radical resection of gastric cancer. BMC Cancer. (2019) 19:833. 10.1186/s12885-019-6024-3 31443699 PMC6708212

[16] Galata C Blank S Weiss C Ronellenfitsch U Reissfelder C Hardt J. Role of Postoperative Complications in Overall Survival after Radical Resection for Gastric Cancer: A Retrospective Single-Center Analysis of 1107 Patients. Cancers. (2019) 11:1890. 10.3390/cancers11121890 31783704 PMC6966624

[17] Brenkman HJF Gisbertz SS Slaman AE Goense L Ruurda JP van Berge Henegouwen MI. Postoperative outcomes of minimally invasive gastrectomy versus open gastrectomy during the early introduction of minimally invasive gastrectomy in the netherlands: a population-based cohort study. Ann Surg. (2017) 266:831–8. 10.1097/SLA.0000000000002391 28742708

[18] Wu L Ge L Qin Y Huang M Chen J Yang Y. Postoperative morbidity and mortality after neoadjuvant chemotherapy versus upfront surgery for locally advanced gastric cancer: a propensity score matching analysis. Cancer Manag Res. (2019) 11:6011–8. 10.2147/CMAR.S203880 31308742 PMC6614824

[19] Ramachandra Goel V Raju K Rao TS Patnaik Nusrath. Prospective randomized controlled study comparing primary surgery versus neoadjuvant chemotherapy followed by surgery in gastric carcinoma. Indian J Surg Oncol. (2019) 10:245–50. 10.1007/s13193-019-00908-7 31168243 PMC6527645

[20] Cuschieri A Fayers P Fielding J Craven J Bancewicz J Joypaul V. Postoperative morbidity and mortality after D1 and D2 resections for gastric cancer: preliminary results of the MRC randomised controlled surgical trial. The surgical cooperative group. Lancet (London, England). (1996) 347:995–9. 10.1016/S0140-6736(96)90144-0 8606613

[21] Zhou J Yu P Shi Y Tang B Hao Y Zhao Y. Evaluation of Clavien-Dindo classification in patients undergoing total gastrectomy for gastric cancer. Med Oncol. (2015) 32:120. 10.1007/s12032-015-0573-3 25788033

[22] Tu RH Lin JX Li P Xie JW Wang JB Lu J. Prognostic significance of postoperative pneumonia after curative resection for patients with gastric cancer. Cancer Med. (2017) 6:2757–65. 10.1002/cam4.1163 29076260 PMC5727328

[23] Terashima M Iwasaki Y Mizusawa J Katayama H Nakamura K Katai H. Randomized phase III trial of gastrectomy with or without neoadjuvant S-1 plus cisplatin for type 4 or large type 3 gastric cancer, the short-term safety and surgical results: Japan Clinical Oncology Group Study (JCOG0501). Gastric Cancer. (2019) 22:1044–52. 10.1007/s10120-019-00941-z 30827001

[24] Tanaka Y Kunisaki C Izumisawa Y Makino H Kimura J Sato S. A phase I/II study of NAC with docetaxel, cisplatin, and S-1 for stage III gastric cancer. Anticancer Res. (2018) 38:6015–21. 10.21873/anticanres.12951 30275234

[25] Yoshikawa T Rino Y Yukawa N Oshima T Tsuburaya A Masuda M. Neoadjuvant chemotherapy for gastric cancer in Japan: a standing position by comparing with adjuvant chemotherapy. Surg Today. (2014) 44:11–21. 10.1007/s00595-013-0529-1 23508452

[26] Yoshikawa T Morita S Tanabe K Nishikawa K Ito Y Matsui T. Survival results of a randomised two-by-two factorial phase II trial comparing neoadjuvant chemotherapy with two and four courses of S-1 plus cisplatin (SC) and paclitaxel plus cisplatin (PC) followed by D2 gastrectomy for resectable advanced gastric cancer. Eur J Cancer. (2016) 62:103–11. 10.1016/j.ejca.2016.04.012 27244537

