# Supplementary material for: Correlative Analysis Between Adverse Events of Preoperative Chemotherapy and Postoperative Complications of Gastric Cancer
Source: Front Surg. 2021 Dec 2;8:768243. doi: 10.3389/fsurg.2021.768243 (PMC8674724; doi:10.3389/fsurg.2021.768243)
Supplement: Supplementary file 1 [file Data_Sheet_1.docx]

Table 1 Baseline characteristic

| Variable | Control (N=266) | NACT (N=77) | SUM (N=343) | *P* value |
| --- | --- | --- | --- | --- |
| Gender |  |  |  | 0.062 |
| Male | 170 (63.9%) | 58 (75.3%) | 228 (66.5%) |  |
| Female | 96 (36.1%) | 19 (24.7%) | 115 (33.5%) |  |
| Age |  |  |  | 0.729 |
| <60 years | 115 (43.2%) | 35 (45.5%) | 150 (43.7%) |  |
| ≥60 years | 151 (56.8%) | 42 (54.5%) | 193 (56.3%) |  |
| City |  |  |  | 0.178 |
| Beijing | 72 (27.1%) | 15 (19.5%) | 87 (25.4%) |  |
| Other cities | 194 (72.9%) | 62 (80.5%) | 256 (74.6%) |  |
| BMI (kg/m^2^) |  |  |  | 0.188 |
| [18.5，25) | 172 (64.7%) | 58 (75.3%) | 230 (67.1%) |  |
| <18.5 | 11 (4.1%) | 2 (2.6%) | 13 (3.8%) |  |
| [25，30) | 77 (28.9%) | 14 (18.2%) | 91 (26.5%) |  |
| ≥30 | 6 (2.3%) | 3 (3.9%) | 9 (2.6%) |  |
| Preoperative comorbidities |  |  |  | 0.194 |
| No | 191 (71.8%) | 61 (79.2%) | 252 (73.5%) |  |
| Yes  NRS-2002  1-2  3-5  ECOG  0  1-3  KPS  100  ≤90  ASA score  I  II-III | 75 (28.2%)  239 (89.8%)  14 (5.3%)  175 (65.8%)  72 (27.1%)  98 (36.8%)  149 (56.0%)  23 (8.7%)  243 (91.4%) | 16 (20.8%)  74 (96.1%)  0 (00.0%)  55 (71.4%)  19 (24.7%)  27 (35.1%)  47 (61.0%)  6 (7.8%)  71 (92.2%) | 91 (26.5%)  313 (91.3%)  14 (4.1%)  230 (67.1%)  91 (26.5%)  125 (36.5%)  196 (57.1%)  29 (8.5%)  314 (91.6%) | 0.186  0.561  0.622  0.812 |
| Smoking history |  |  |  | 0.068 |
| No | 185 (69.5%) | 45 (58.4%) | 230 (67.1%) |  |
| Yes | 81 (30.5%) | 32 (41.6%) | 113 (32.9%) |  |
| Drinking history |  |  |  | 0.680 |
| No | 187 (70.3%) | 56 (72.7%) | 243 (70.8%) |  |
| Yes | 79 (29.7%) | 21 (27.3%) | 100 (29.2%) |  |
| Family history (cancer) |  |  |  | 0.226 |
| No | 245 (92.1%) | 74 (96.1%) | 319 (93.0%) |  |
| Yes | 21 (7.9%) | 3 (3.9%) | 24 (7.0%) |  |
| Family history (other disease) |  |  |  | 0.665 |
| No | 241 (90.6%) | 71 (92.2%) | 312 (91.0%) |  |
| Yes | 25 (9.4%) | 6 (7.8%) | 31 (9.0%) |  |

eTable 2 Perioperative information

| Variable | Control (N=266) | NACT (N=77) | SUM (N=343) | *P* value |
| --- | --- | --- | --- | --- |
| Operating time |  |  |  | 0.007 |
| <180min | 111 (41.7%) | 19 (24.7%) | 130 (37.9%) |  |
| ≥180min | 155 (58.3%) | 58 (75.3%) | 213 (62.1%) |  |
| Preoperative T stage |  |  |  | <0.001 |
| 1 | 55 (20.7%) | 0 (0%) | 55 (16.0%) |  |
| 2 | 33 (12.4%) | 2 (2.6%) | 35 (10.2%) |  |
| 3 | 79 (29.7%) | 34 (44.2%) | 113 (32.9%) |  |
| 4a | 80 (30.1%) | 36 (46.8%) | 116 (33.8%) |  |
| 4b | 19 (7.1%) | 5 (6.5%) | 24 (7.0%) |  |
| Preoperative N stage |  |  |  | <0.001 |
| 0 | 120 (45.1%) | 10 (13.0%) | 130 (37.9%) |  |
| 1 | 75 (28.2%) | 17 (22.1%) | 92 (26.8%) |  |
| 2 | 46 (17.3%) | 37 (48.1%) | 83 (24.2%) |  |
| 3 | 25 (9.4%) | 13 (16.9%) | 38 (11.1%) |  |
| Preoperative M stage |  |  |  | 0.418 |
| 0 | 246 (92.5%) | 69 (89.6%) | 315 (91.8%) |  |
| 1 | 20 (7.5%) | 8 (10.4%) | 28 (8.2%) |  |
| Tumor Location |  |  |  | 0.115 |
| L | 141 (53.0%) | 32 (41.6%) | 173 (50.4%) |  |
| EGJ | 40 (15.0%) | 22 (28.6%) | 62 (18.1%) |  |
| U | 28 (10.5%) | 8 (10.4%) | 36 (10.5%) |  |
| M | 55 (20.7%) | 14 (18.2%) | 69 (20.1%) |  |
| Total gastric | 2 (0.8%) | 1 (1.3%) | 3 (0.9%) |  |
| Surgical procedure |  |  |  | 0.619 |
| Laparotomy | 118 (44.4%) | 39 (50.6%) | 157 (45.8%) |  |
| Laparoscopic | 146 (54.9%) | 38 (49.4%) | 184 (53.6%) |  |
| Thoracoabdominal | 2 (0.8%) | 0 (0.0%) | 2 (0.6%) |  |
| Resection range |  |  |  | <0.001 |
| Distal | 175 (65.8%) | 35 (45.5%) | 210 (61.2%) |  |
| Proximal | 25 (9.4%) | 5 (6.5%) | 30 (8.7%) |  |
| Total | 66 (24.8%) | 37 (48.1%) | 103 (30.0%) |  |
| Multiorgan excision |  |  |  | 0.170 |
| No | 245 (92.1%) | 67 (87.0%) | 312 (91.0%) |  |
| Yes | 21 (7.9%) | 10 (13.0%) | 31 (9.0%) |  |
| Lymph node dissection range |  |  |  | 0.248 |
| D2 | 248 (93.2%) | 76 (98.7%) | 324 (94.5%) |  |
| D1+ | 13 (4.9%) | 1 (1.3%) | 14 (4.1%) |  |
| D1 | 5 (1.9%) | 0 (0.0%) | 5 (1.5%) |  |
| Surgical radicalness |  |  |  | 0.256 |
| R0 | 255 (95.9%) | 77 (100.0%) | 332 (96.8%) |  |
| R1 | 9 (3.4%) | 0 (0.0%) | 9 (2.6%) |  |
| R2 | 2 (0.8%) | 0 (0.0%) | 2 (0.6%) |  |
| Reconstruction approach |  |  |  | 0.007 |
| Billroth I | 23 (8.6%) | 1 (1.3%) | 24 (7.0%) |  |
| Billroth II | 153 (57.5%) | 37 (48.1%) | 190 (55.4%) |  |
| Others | 90 (33.8%) | 39 (50.6%) | 129 (37.6%) |  |
| Complications |  |  |  | 0.196 |
| No | 180 (67.7%) | 46 (59.7%) | 226 (65.9%) |  |
| Yes | 86 (32.3%) | 31 (40.3%) | 117 (34.1%) |  |

eTable 3.1 Univariate analysis of postoperative complications

| Variable | No complications (N=226) | Complications (N=117) | OR (95%CI) | *P* Value |
| --- | --- | --- | --- | --- |
| Gender |  |  |  |  |
| Male | 149 | 77 | Ref |  |
| Female | 79 | 38 | 1.074 (0.668-1.727) | 0.767 |
| Age |  |  |  |  |
| <60 years | 105 | 45 |  |  |
| ≥60 years | 121 | 72 | 1.388 (0.881-2.188) | 0.157 |
| BMI |  |  |  |  |
| [18.5，25) | 153 | 77 | Ref |  |
| <18.5 | 9 | 4 | 0.883 (0.264-2.959) | 0.840 |
| [25，30) | 58 | 33 | 1.131 (0.681-1.878) | 0.636 |
| ≥30 | 6 | 3 | 0.994 (0.242-4.080) | 0.993 |
| Area |  |  |  |  |
| Beijing | 51 | 36 | Ref |  |
| Other cities | 175 | 81 | 0.656 (0.397-1.083) | 0.099 |
| Preoperative comorbidities |  |  |  |  |
| No | 175 | 77 | Ref |  |
| Yes | 51 | 40 | 1.783 (1.089-2.919) | 0.022 |
| NRS-2002  1-2  3-5  ECOG  0  1-3  KPS  100  ≤90  ASA score  I  II-III  TRG classifications  0-1  2-3 | 205  9  152  56  81  130  19  207  9  34 | 107  7  78  35  44  66  10  107  11  31 | Ref  0.671 (0.243-1.852)  Ref  0.821 (0.497-1.358)  Ref  1.070 (0.668-1.715)  Ref  0.457 (0.161-1.300)  Ref  0.138 | 0.441  0.442  0.779  0.138  0.184 |
| Family history (cancer) |  |  |  |  |
| No | 209 | 110 | Ref |  |
| Yes | 17 | 7 | 0.782 (0.315-1.944) | 0.597 |
| Family history (other disease) |  |  |  |  |
| No | 206 | 106 | Ref |  |
| Yes | 20 | 11 | 1.069 (0.494-2.313) | 0.866 |
| Smoking history |  |  |  |  |
| No | 153 | 77 | Ref |  |
| Yes | 73 | 40 | 1.089 (0.679-1.747) | 0.724 |
| Drinking history |  |  |  |  |
| No | 161 | 82 | Ref |  |
| Yes | 65 | 35 | 1.057 (0.648-1.725) | 0.824 |
| Tumor Location |  |  |  |  |
| L | 119 | 54 | Ref |  |
| EGJ | 36 | 26 | 1.592 (0.875-2.895) | 0.128 |
| U | 26 | 10 | 0.848 (0.382-1.881) | 0.684 |
| M | 43 | 26 | 1.332 (0.743-2.388) | 0.335 |
| Total gastric | 2 | 1 | 1.102 (0.098-12.415) | 0.937 |
| Preoperative T stage |  |  |  |  |
| 1 | 37 | 18 | Ref |  |
| 2 | 26 | 9 | 0.712 (0.277-1.829) | 0.480 |
| 3 | 80 | 33 | 0.848 (0.424-1.697) | 0.641 |
| 4a | 74 | 42 | 1.167 (0.582-2.300) | 0.656 |
| 4b | 9 | 15 | 3.426 (1.260-9.313) | 0.016 |
| Preoperative N stage |  |  |  |  |
| 0 | 91 | 39 | Ref |  |
| 1 | 58 | 34 | 1.368 (0.777-2.408) | 0.278 |
| 2 | 55 | 28 | 1.188 (0.659-2.142) | 0.567 |
| 3 | 22 | 16 | 1.697 (0.805-3.576) | 0.164 |
| Preoperative M stage |  |  |  |  |
| 0 | 213 | 102 | Ref |  |
| 1 | 12 | 15 | 2.410 (1.105-5.253) | 0.027 |
| Borrmann typing |  |  |  |  |
| Type I | 28 | 9 | Ref |  |
| Type II | 44 | 25 | 1.768 (0.721-4.336) | 0.213 |
| Type III | 135 | 71 | 1.636 (0.732-3.656) | 0.230 |
| Type IV | 10 | 4 | 1.244 (0.313-4.954) | 0.756 |
| Unknown | 9 | 8 | 2.765 (0.822-9.300) | 0.100 |
| Operating time |  |  |  |  |
| <180min | 90 | 40 | Ref |  |
| ≥180min | 136 | 77 | 1.274 (0.800-2.029) | 0.308 |
| Surgical procedure |  |  |  |  |
| Laparotomy | 98 | 59 | Ref |  |
| Laparoscopic | 126 | 58 | 0.765 (0.488-1.197) | 0.241 |
| Thoracoabdominal | 2 | 0 | 0.000 (0.000-) | 0.999 |
| Resection range |  |  |  |  |
| Distal | 146 | 64 | Ref |  |
| Proximal | 20 | 10 | 1.141 (0.505-2.574) | 0.751 |
| Total | 60 | 43 | 1.635 (1.002-2.668) | 0.049 |
| Anastomosis |  |  |  |  |
| Billroth I | 19 | 5 | Ref |  |
| Billroth II | 131 | 59 | 1.711 (0.610-4.803) | 0.307 |
| Others | 76 | 53 | 2.650 (0.931-7.540) | 0.068 |
| Multiorgan excision |  |  |  |  |
| No | 209 | 103 | Ref |  |
| Yes | 17 | 14 | 1.671 (0.793-3.522) | 0.177 |
| Lymph node dissection range |  |  |  |  |
| D2 | 212 | 112 | Ref |  |
| D1+ | 9 | 5 | 1.052 (0.344-3.213) | 0.930 |
| D1 | 5 | 0 | 0.000 (0.000-) | 0.999 |
| Surgical radicalness |  |  |  |  |
| R0 | 220 | 112 | Ref |  |
| R1 | 5 | 4 | 1.571 (0.414-5.967) | 0.507 |
| R2 | 1 | 1 | 1.964 (0.122-31.698) | 0.634 |
| Preoperative chemotherapy |  |  |  |  |
| No | 180 | 86 | Ref |  |
| Yes | 46 | 31 | 1.411 (0.836-2.379) | 0.197 |

eTable 3.2 Multivariate analysis of postoperative complications in two groups

| Variable | *P* Value | Exp (B) | 95%CI | |
| --- | --- | --- | --- | --- |
|  |  |  | Lower | Upper |
| Area |  |  |  |  |
| Beijing |  | Ref |  |  |
| Others | 0.132 | 0.668 | 0.396 | 1.129 |
| Preoperative comorbidities |  |  |  |  |
| No |  | Ref |  |  |
| Yes | 0.026 | 1.788 | 1.070 | 2.954 |
| Preoperative T stage |  |  |  |  |
| 1 |  | Ref |  |  |
| 2 | 0.469 | 0.701 | 0.268 | 1.835 |
| 3 | 0.398 | 0.732 | 0.355 | 1.509 |
| 4a | 0.908 | 1.042 | 0.515 | 2.107 |
| 4b | 0.028 | 3.163 | 1.130 | 8.853 |
| Preoperative M stage |  |  |  |  |
| 0 |  | Ref |  |  |
| 1 | 0.106 | 0.647 | 0.382 | 1.097 |
| Resection range |  |  |  |  |
| Distal |  | Ref |  |  |
| Proximal | 0.443 | 0.523 | 0.100 | 2.735 |
| Total | 0.457 | 0.574 | 0.133 | 2.473 |
| Anastomosis |  |  |  |  |
| Billroth I |  | Ref |  |  |
| Billroth II | 0.383 | 1.602 | 0.556 | 4.619 |
| Others | 0.074 | 2.678 | 0.909 | 7.889 |
| Preoperative chemotherapy |  |  |  |  |
| No |  | Ref |  |  |
| Yes | 0.206 | 1.448 | 0.816 | 2.568 |

eTable 4 Incidence of adverse events

| Classification | Adverse event classification (CTCAEv5.0) | | |
| --- | --- | --- | --- |
|  | Class 1-2 (%) | Class 3-4 (%) | ALL (%) |
| Blood/marrow system |  |  |  |
| Leukopenia | 26 (33.77%) | 3 (3.90%) | 29 (37.66%) |
| Thrombocytopenia | 23 (29.87%) | 2 (2.60%) | 25 (32.47%) |
| Decreased hemoglobin | 23 (29.87%) | 1 (1.30%) | 24 (31.17%) |
| Neutropenia | 15 (19.48%) | 3 (3.90%) | 18 (23.38%) |
| Other abnormal | 1 (1.30%) | 0 (0%) | 1 (1.30%) |
| Systemic symptoms |  |  |  |
| Fatigue | 64 (83.12%) | 3 (3.90%) | 67 (87.01%) |
| Sweating (night sweats) | 47 (61.04%) | 0 (0%) | 47 (61.04%) |
| Insomnia | 43 (55.84%) | 4 (5.19%) | 47 (61.04%) |
| Weight loss | 41 (53.25%) | 0 (0%) | 41 (53.25%) |
| Fever (no neutropenia) | 4 (5.19%) | 0 (0%) | 4 (5.19%) |
| weight gain | 1 (1.30%) | 0 (0%) | 1 (1.30%) |
| Gastrointestinal system |  |  |  |
| Anorexia | 56 (72.73%) | 3 (3.90%) | 59 (76.62%) |
| Nausea | 45 (58.44%) | 0 (0%) | 45 (58.44%) |
| Constipation | 38 (49.35%) | 1 (1.30%) | 39 (50.65%) |
| Bloating | 38 (49.35%) | 1 (1.30%) | 39 (50.65%) |
| Heartburn/indigestion | 36 (46.75%) | 1 (1.30%) | 37 (48.05%) |
| Change in taste | 36 (48.21%) | 0 (0%) | 36 (48.05%) |
| Diarrhea | 25 (32.47%) | 2 (2.60%) | 27 (35.06%) |
| Dysphagia | 17 (22.08%) | 1 (1.30%) | 18 (17.86%) |
| Mucositis/stomatitis | 11 (14.29%) | 2 (2.60%) | 13 (16.88%) |
| Vomiting | 15 (19.48%) | 0 (0%) | 15 (19.48%) |
| Skin change |  |  |  |
| Dry skin | 39 (50.65%) | 0 (0%) | 39 (50.65%) |
| Pigmentation | 38 (49.35%) | 0 (0%) | 38 (49.35%) |
| Pruritus | 36 (46.75%) | 0 (0%) | 36 (46.75%) |
| Rash peeling | 17 (22.08%) | 1 (1.30%) | 18 (23.38%) |
| Hypopigmentation | 18 (23.38%) | 0 (0%) | 18 (23.38%) |
| Hair loss/balding | 17 (22.08%) | 0 (0%) | 17 (22.08%) |
| Flushing | 14 (18.18%) | 0 (0%) | 14 (18.18%) |
| Nail changes | 10 (12.99%) | 0 (0%) | 10 (12.99%) |
| Metabolism/Laboratory examination |  |  |  |
| Abnormal ALT | 9 (11.69%) | 1 (1.30%) | 10 (12.99%) |
| Abnormal AST | 9 (11.69%) | 0 (0%) | 9 (11.69%) |
| Hypoproteinemia | 7 (9.09%) | 0 (0%) | 7 (9.09%) |
| Hypertriglyceridemia | 5 (6.49%) | 0 (0%) | 5 (6.49%) |
| Abnormal γ-glutamyl transferase | 4 (5.19%) | 1 (1.30%) | 5 (6.49%) |
| Hyperuricemia | 4 (5.19%) | 0 (0%) | 4 (5.19%) |
| Hyperbilirubinemia | 3 (3.90%) | 0 (0%) | 3 (3.90%) |
| Hyperglycemia | 1 (1.30%) | 0 (0%) | 1 (1.30%) |
| Abnormal creatinine | 1 (1.30%) | 0 (0%) | 1 (1.30%) |
| Abnormal alkaline phosphatase | 1 (1.30%) | 0 (0%) | 1 (1.30%) |
| Acidosis | 1 (1.30%) | 0 (0%) | 1 (1.30%) |
| Other abnormal | 1 (1.30%) | 0 (0%) | 1 (1.30%) |
| Hepatopancreas |  |  |  |
| Abnormal hepatopancreas | 1 (1.30%) | 0 (0%) | 1 (1.30%) |
| Nervous system |  |  |  |
| Sensory neuropathy | 48 (62.34%) | 4 (5.19%) | 52 (67.53%) |
| Pain |  |  |  |
| Pain | 45 (58.44%) | 1 (1.30%) | 46 (59.74%) |
| Musculoskeletal/soft tissue |  |  |  |
| Arthritis | 35 (45.45%) | 0 (0%) | 35 (45.45%) |
| Total AEs | 55 (71.4%) | 22 (28.6%) | 77 (100.0%) |

eTable 5 Association between adverse events and postoperative complications

| Classification | No complications (N=46) | Complications (N=31) | SUM  (N=77) | *P* Value |
| --- | --- | --- | --- | --- |
| Blood/marrow system |  |  |  | 0.970 |
| No | 18 (39.1%) | 12 (38.7%) | 30 (39.0%) |  |
| Yes | 28 (60.9%) | 19 (61.3%) | 47 (61.0%) |  |
| Systemic symptoms |  |  |  | 0.801 |
| No | 2 (4.3%) | 1 (3.2%) | 3 (3.9%) |  |
| Yes | 44 (95.7%) | 30 (96.8%) | 74 (96.1%) |  |
| Gastrointestinal system |  |  |  | >0.999 |
| No | 3 (6.5%) | 2 (6.5%) | 5 (6.5%) |  |
| Yes | 43 (93.5%) | 29 (93.5%) | 72 (93.5%) |  |
| Skin change |  |  |  | 0.635 |
| No | 7 (15.2%) | 6 (19.4%) | 13 (16.9%) |  |
| Yes | 39 (84.8%) | 25 (80.6%) | 64 (83.1%) |  |
| Metabolism/Laboratory examination |  |  |  | 0.895 |
| No | 32 (69.6%) | 22 (71.0%) | 54 (70.1%) |  |
| Yes | 14 (30.4%) | 9 (29.0%) | 23 (29.9%) |  |
| Hepatopancreas |  |  |  | 0.403 |
| No | 46 (100%) | 20 (96.8%) | 76 (98.7%) |  |
| Yes | 0 (0%) | 1 (3.2%) | 1 (1.3%) |  |
| Nervous system |  |  |  | 0.740 |
| No | 15 (32.6%) | 9 (29.0%) | 24 (31.2%) |  |
| Yes | 31 (67.4%) | 22 (71.0%) | 53 (68.8%) |  |
| Pain |  |  |  | 0.806 |
| No | 18 (39.1%) | 13 (41.9%) | 31 (40.3%) |  |
| Yes | 28 (60.9%) | 18 (58.1%) | 46 (59.7%) |  |
| Musculoskeletal/soft tissue |  |  |  | 0.818 |
| No | 24 (52.2%) | 17 (54.8%) | 41 (53.2%) |  |
| Yes | 22 (47.8%) | 14 (45.2%) | 36 (46.8%) |  |
